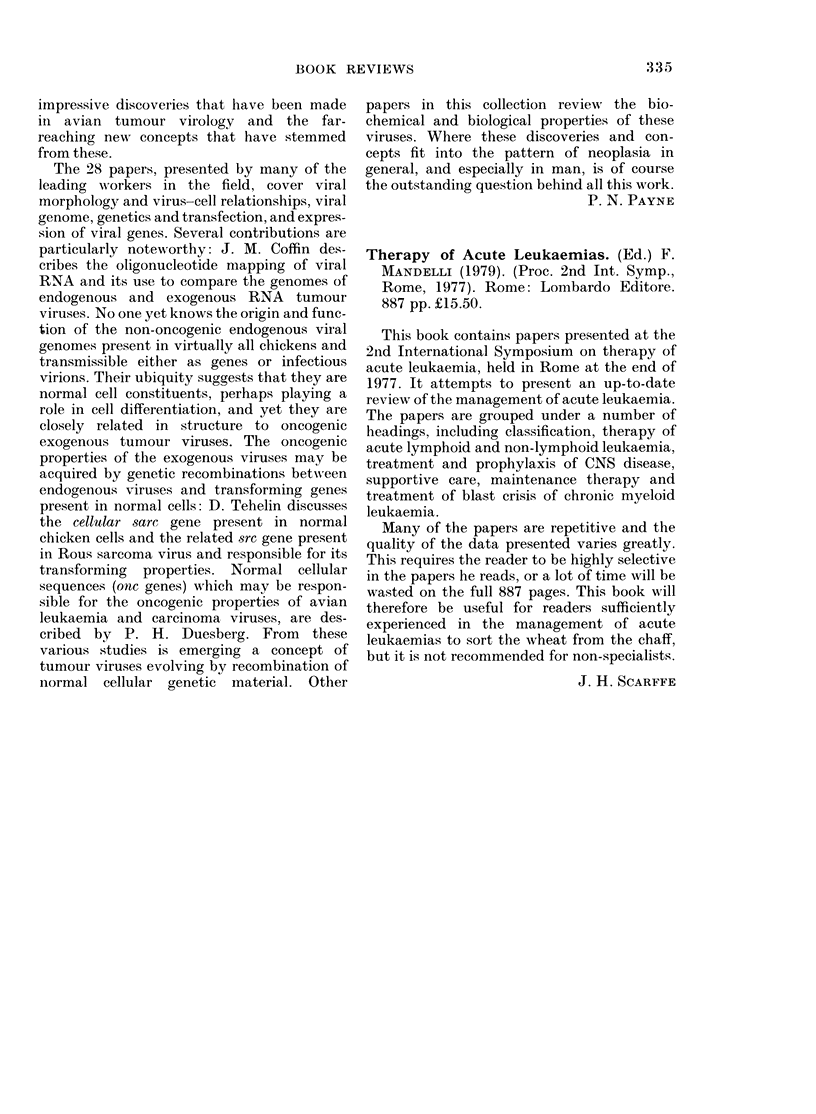# Therapy of Acute Leukaemias

**Published:** 1980-02

**Authors:** J. H. Scarffe


**Therapy of Acute Leukaemias.** (Ed.) F. MANDELLI (1979). (Proc. 2nd Int. Symp., Rome, 1977). Rome: Lombardo Editore. 887 pp. £15.50.

This book contains papers presented at the 2nd International Symposium on therapy of acute leukaemia, held in Rome at the end of 1977. It attempts to present an up-to-date review of the management of acute leukaemia. The papers are grouped under a number of headings, including classification, therapy of acute lymphoid and non-lymphoid leukaemia, treatment and prophylaxis of CNS disease, supportive care, maintenance therapy and treatment of blast crisis of chronic myeloid leukaemia.

Many of the papers are repetitive and the quality of the data presented varies greatly. This requires the reader to be highly selective in the papers he reads, or a lot of time will be wasted on the full 887 pages. This book will therefore be useful for readers sufficiently experienced in the management of acute leukaemias to sort the wheat from the chaff, but it is not recommended for non-specialists.

J. H. SCARFFE